# Combination Therapy of Interferon Beta-1b and Tacrolimus: A Pilot Safety Study

**DOI:** 10.1155/2012/935921

**Published:** 2012-08-15

**Authors:** F. Jacques, I. Gaboury, S. Christie, F. Grand'Maison

**Affiliations:** ^1^Division of Neurology, CSSSG-Hull Pavillion, Clinique Neuro-Outaouais, 147 Boulevard d'Europe, Gatineau, QC, Canada J9J A05; ^2^Research Institute, Children's Hospital of Eastern Ontario, 401 Smyth Road, Ottawa, ON, Canada K1H 8L1; ^3^Division of Neurology, Ottawa Hospital, Nepean Medical Center, 1 Centrepointe Drive, Suite 407, Ottawa, ON, Canada K2G 6E2; ^4^Division of Neurology, Charles Lemoynes Hospital Montreal, Clinique Neuro Rive-Sud, 4896 Boulevard Taschereau, Suite 250, Greenfield Park, QC, Canada J4V 2J2

## Abstract

Tacrolimus is a calcineurin inhibitor which works to induce immune suppression by preventing cytokine transcription and lymphocyte activation. Combining the immunomodulator interferon beta-1b (Betaseron) with the immunosuppressant tacrolimus (Prograf) may have the potential of additive therapeutic benefit through the complementary mechanisms of action of these two therapeutics. In this randomized, open-label, multicenter, two-arm pilot study, the authors examined the safety and tolerability of the combination of interferon beta-1b and tacrolimus in relapsing remitting (RRMS) and secondary progressive (SPMS) multiple sclerosis patients who have failed one or more immunomodulatory therapies. Patients (*n* = 25) received a combination of interferon beta-1b subcutaneously every other day and oral tacrolimus (low blood level tacrolimus, 1–5 ng/mL, or high blood level tacrolimus, 5–10 ng/mL) for a period of 38 weeks. The combination therapy of interferon beta-1b and tacrolimus over the 10-month period of the study was shown to be safe and relatively well tolerated. There were no unexpected adverse events occurring as the result of the combination therapy. Further study of this combination therapy in patients with multiple sclerosis unresponsive to conventional therapy is warranted.

## 1. Introduction

The autoimmune pathophysiological process by which the immune system carries out its attack of the central nervous system in multiple sclerosis (MS) is complex and multipronged and can vary between patients and even within the same patient, over time [1]. As in all other autoimmune diseases such as rheumatoid arthritis, lupus, and myasthenia gravis, among others, there is not a single therapy that will be effective for all patients of that same disease.

More than 15 years of experience with current immunomodulators, when used as monotherapy in multiple sclerosis, has shown that their clinical efficacy is only modest, and that new therapeutic strategies are required. The more recent therapies of natalizumab and fingolimod, though more effective than conventional immunomodulators, each carry higher risks of serious side effects. Combination therapies in MS may have the potential of offering additive benefit through to patients with MS by targeting multiple autoimmune processes. As previously described with respect to MS, combination therapy with drugs utilizing complementary mechanisms of action [2] may have the potential for improving disease control by targeting multiple inflammatory or autoimmune aspects of the disease at once. Interferon beta-1b (Betaseron) was the first clinically effective therapeutic agent proven to modify the disease course in MS patients and is thought to exert its therapeutic effects by downregulating expression of MHC II molecules and inhibiting the passage of immune cells into the CNS [3–6]. Tacrolimus (FK506) is a macrolide isolated from *Streptomyces tsukubaensis* and is currently used as an immunosuppressant to prevent allograft rejection in transplant patients [7]. Tacrolimus exerts its immunosuppressive effect by binding to the FK506 binding protein-12 (FKBP-12) in lymphocytes and inhibiting calcineurin activation, resulting in reduced cytokine production (IL-2) and lymphocyte activation [8–12]. Tacrolimus has also been shown to have neuroprotective or neuroregenerative effects in models of nerve crush, nerve transection, and spinal cord injury [13–16]. It has also been shown to prevent induction of myasthenia gravis in a rat model [17].

In a model of chronic experimental autoimmune encephalomyelitis (EAE), tacrolimus demonstrated clinical improvement as well as reduced demyelination and axonal loss [14]. The latter changes could also be achieved using nonimmunosuppressant doses of tacrolimus, thus suggesting alternative mechanisms of action other than immunosuppression, such as neuroprotection and/or neuroregeneration. This has been attributed to binding of the FK-52 binding protein, a component of the mature steroid receptor complex [13, 14]. Previous studies have evaluated the safety and tolerability of tacrolimus in secondary progressive multiple sclerosis (SPMS) patients [18].

Taken together, interferon beta-1b and tacrolimus are two therapeutic agents which exert their immunomodulatory effects by similar but distinct mechanisms. Such combination therapy may result in improved treatment outcomes for relapsing remitting multiple sclerosis (RRMS) and secondary progressive (SPMS) patients who have failed one or more previous immunomodulating therapies. Furthermore, previous studies suggest that combination therapies may result in improved treatment adherence rates [2]. As such, we believe that exploring their potential use in combination therapy is warranted and may represent an effective therapeutic option for patients with MS.

In this study, we examined the safety and tolerability of the combination therapy consisting of interferon beta-1b and tacrolimus in relapsing remitting multiple sclerosis (RRMS) and secondary progressive (SPMS) patients who have failed one or more previous immunomodulating therapies.

## 2. Objectives

The primary objective was to evaluate the safety and tolerability of a combination of interferon beta-1b and tacrolimus in patients with RRMS or SPMS who have failed previous treatment with approved disease-modifying agents. Treatment failure was defined as having the same or higher annual relapse rate, or sustained progression of ≥1.0 point on the expanded disability status scale (EDSS) score for 3 months, despite immunomodulatory treatment, for at least six months.

## 3. Study Design and Methods

**Figure 1:**
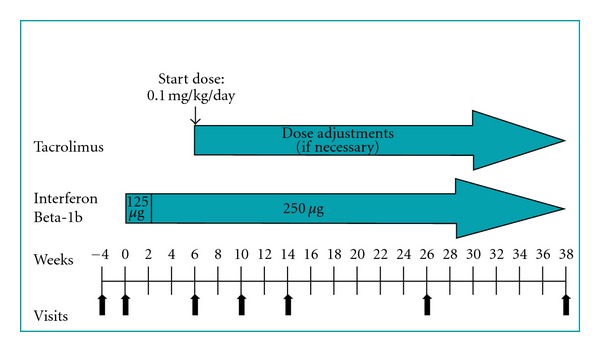
Study design. Relapse remitting and secondary progressive patients were randomized into low-blood-level (LBL) or high-blood-level (HBL) tacrolimus groups, in combination with Betaseron. Betaseron was administered subcutaneously every other day, while tacrolimus was given orally. Combination therapy was administered over a 38-week period.

This study was a randomized, open-label, evaluator-blinded, multicenter, two-arm, parallel group, 38-week phase IIa pilot study to evaluate the safety and tolerability of interferon beta-1b, 250 *μ*g (8 MIU) administered subcutaneously every other day in combination with tacrolimus (Figure 1). Tacrolimus was administered orally twice daily 12 hours apart on an empty stomach, at a dose to reach either low (1–5 ng/mL) or high (5–10 ng/mL) whole blood concentrations, hereafter designated as LBL group (low blood level) and HBL group (high blood level) [19]. Study ethics approval was granted by the local ethics committees of the CSSSG—Hull Hospital in Gatineau and the Charles LeMoyne Hospital in Montreal—and by an independent committee, IRB Services (Aurora, Ontario, Canada). All patients gave written informed consent prior to their participation in the study. Patients eligible for randomization were required to be 18 to 55 years of age and have a diagnosis of MS according to the McDonald criteria [20] and an EDSS score of less than seven. Only patients who had failed treatment with currently approved disease-modifying drugs (DMDs) for MS were recruited. DMDs for which patients had prior treatment failure include Betaseron, Avonex, Rebif, and Copaxone. This clinical study was registered with the US National Institutes of Health on http://www.clinicaltrials.gov/ (trial NCT00298662).

### 3.1. Randomization and Blinding

Patients were enrolled by the treating neurologist, who was not involved in the patient group assignment. Patient allocation was done by a research nurse using a computer-generated randomization sheet provided by Berlex Canada. Once a participant was enrolled, the next sequential treatment assignment was used for the allocation.

### 3.2. Intervention

Patients started therapy with interferon beta-1b at half the standard dose (125 *μ*g) [4 MIU] every other day for 2 weeks, prior to escalating to the fixed 250 *μ*g dose every other day. All patients received injection training [21].

Tacrolimus therapy was initiated at week 6 (or when full-dose interferon beta-1b was tolerated) at a starting dose of 0.1 mg/kg/day administered twice daily. The dose was adjusted in order to reach the desired low or high blood concentration range (dose adjustments took from 2 to 4 weeks) and was evaluated monthly to ensure that the level remained in the assigned range. The use of corticosteroids (e.g., intravenous methylprednisolone 1000 mg/day for 3 days) was allowed for the treatment of acute relapses.

Safety and tolerability was assessed with physical examinations, laboratory evaluations, and recording of adverse events. The latter were collected at each clinic visit as shown in Table 1. Additional parameters including relapse rate, EDSS, and visual analogue scale evaluations were done every three months. The number of T1 and T2 lesions, as well as T1 gadolinium enhancing lesions, was evaluated by blinded MRI upon study entry and exit. Multiple sclerosis functional composite (MSFC) and EDSS evaluation and scoring were done by an examiner blinded to the group assignment.

### 3.3. Statistical Analysis

All statistical analyses were performed according to the intention-to-treat principle. Descriptive statistics of baseline data for both groups are summarized in Table 2. Unless otherwise mentioned, data presented are medians and range, or interquartile range and frequencies. Comparisons between study groups were performed using a Wilcoxon Mann-Whitney test for nonparametric distributions. When statistical differences were assessed between baseline and end of study within a study group, Wilcoxon signed-rank tests were used. Two-sided *P* values of <0.05 were considered to be statistically significant.

## 4. Results

**Figure 2:**
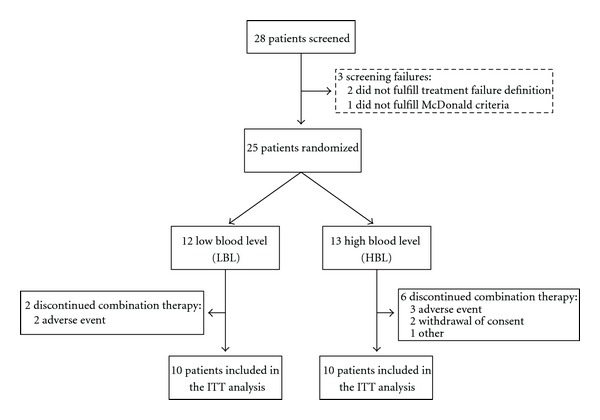
Patient enrollment flow chart. 25 patients who had met all required enrolment criteria were randomly assigned to receive low-blood-level tacrolimus (LBL) or high-blood-level tacrolimus (HBL). 10 LBL patients and 10 HBL patients completed the study and were included in the ITT analysis.

This randomized, open-label, multicenter, two-arm dosage trial took place between February 2003 and October 2006 at three MS clinics in Canada. The flow of participants through the different stages of the trial is shown in Figure 2. There were no significant protocol deviations.

Baseline patient characteristics of both groups are shown in Table 2. Though the HBL group had a greater percentage of female and RRMS patients with a higher baseline RR than the LBL group, none of these differences achieved statistical significance. All patients had previously failed, as per protocol definition, one or more immunomodulating therapies.

All adverse events (AEs) were recorded for all patients. Five total patients dropped out of the pilot study. One patient was removed due to a revised diagnosis of MS. Combination therapy-related AEs resulted in three patients discontinuing combination therapy but continued on monotherapy (2 continued on interferon beta-1b, 1 continued on tacrolimus). One patient completely discontinued all therapy. Two patients were lost to followup.

Twenty patients completed the study in an intention to treat manner. There were no unexpected AEs occurring as the result of the combination. Eighteen different AEs were noted in more than 10% of patients (see Table 3). Of those, injection site reactions, headaches, and flu-like symptoms were the most common. There were no significant differences between groups in incidence of AEs, except for injection site reactions which were more frequent in LBL group and hypomagnesemia in the HBL group. Headaches were more frequent in the HBL group, but this did not reach statistical significance. AEs for which patients withdrew from therapy (*n* = 3) included hyperglycemia, headache, injection site reactions, tremor, and diarrhea.

No significant increase in baseline values of mean creatinine or glucose was seen in either group. A mild decrease was noted in mean magnesium and platelets levels in both groups. No clinically significant differences in lab values were noted between groups over the 10-month period. The mean tacrolimus level during the study was 4.68 ng/mL (sd 0.90) in the LBL group and 7.63 ng/mL (sd 1.44) in the HBL group.

Only the HBL group showed an increase (9.1 mmHg) in mean end-of-study systolic blood pressure when compared to baseline and was considered to be significant when compared to the LBL group (*P* = 0.013).

No therapy-related serious adverse event (SAE) occurred. There were no deaths, and no permanent morbidity was noted for any patient.

Only the LBL group showed an improvement in quality of life as measured by the median change from baseline in the visual analogue scale (VAS) (median improvement of 15 mm, [range −58, 43] in the LBL group versus −1 mm [range −18, 35]) for the HBL group. However, this difference between groups was not found to be statistically significant (*P* = 0.089).

## 5. Discussion

This pilot safety study is the first to describe the safety and tolerability of the combination of interferon beta-1b and tacrolimus in MS patients.

Combination therapy can result in an additive or even synergistic effect in benefit and/or increased incidence of adverse events (AEs). The combination therapy did not generate any untoward drug interactions or unexpected side effects. There was no evidence of opportunistic infection or impaired immune surveillance. The lack of tacrolimus-related pancreatic or renal dysfunction is reassuring and likely explained by the lower blood levels of tacrolimus attained as compared to transplant trials; however, a longer duration of study is needed to confirm this.

The study withdrawal rate of 20% (95% CI: 8.9%, 39.1%) was higher than the 13% obtained in the 8 MIU arm of the interferon beta-1b pivotal trial [4]. 1/5 patients withdrew from the study due to a revised diagnosis of MS, making them ineligible to continue participating in this pilot study. However, 3/5 patients did continue on either interferon beta-1b (*n* = 2) or tacrolimus (*n* = 1) monotherapy, and there was no patient withdrawal as a result of perceived lack of efficacy or due to combination therapy-associated adverse events. Discounting the patient withdrawn due to a revised diagnosis of MS from would adjust the withdrawal rate to 16%, thus brining it closer in line with previous findings [4]. Overall, we believe that the patient withdrawal rate appears magnified due to the small patient sample size enrolled in this pilot study.

Some side effects were more frequently found in the HBL group (headache, hypomagnesemia, and increase in systolic blood pressure), but interestingly, injection site reactions were significantly lower in the HBL group possibly as a result of the higher dose of tacrolimus. Otherwise, no other difference was found in the incidence or type of AE between groups. The lack of difference between groups, however, may be explained by the small size of the cohort in this study.

Several patients continued on the combination therapy initially through a controlled one-year extension (unpublished data) and then in an uncontrolled fashion for several additional years (some up to 8 years). Within that small group's uncontrolled long-term experience, no additional side effects other than what has been previously described in this study occurred, and specifically no significant increase in blood pressure or occurrence of opportunistic infections or cancer was observed (unpublished observations).

Lastly, it was observed that both tacrolimus treatment groups experienced a nonsignificant decrease in EDSS over the course of the study, and MRI scan results indicated a median number of T2 and T1 lesions per scan remaining unchanged over the duration of the study (data not shown). While this pilot study was not designed to evaluate efficacy, we believe these findings lend support to the safety profile of this particular combination therapy. Future studies with larger patient cohorts will be designed to expand on these observations and to answer questions regarding the clinical efficacy of combination therapy.

Overall, results of this pilot safety study suggest that further studies with larger numbers and longer duration may be warranted. An add-on approach rather than a complete switch may prove to be more beneficial for some patients. The combination of interferon beta-1b and tacrolimus may potentially be considered as a therapeutic option for patients with aggressive disease unresponsive to conventional DMD agents, provided, as for all combination therapies in general, close monitoring and predefined efficacy goals and safety stopping rules are in place.

## Figures and Tables

**Table 1:** Schedule of events for each clinic visit.

Visits	1	2	3	4	5	6	7	Extra procedures
	Screening day -30 to -1	Baseline day 1	Week 6^d^	Week 10	Week 14	Week 26	Week 38 final visit	W8, 12, 16, 20, 24, 28, 32
Safety assessment								
Physical exam	*√*	*√*	*√*	*√*	*√*	*√*	*√*	
Vital signs	*√*	*√*	*√*	*√*	*√*	*√*	*√*	
Weight	*√*	*√*	*√*	*√*	*√*	*√*	*√*	
Laboratory	*√*	*√* ^ a^	*√*				*√*	*√*
Serum beta-HCG	*√*	*√* ^ a^	*√*	*√*	*√*	*√*	*√*	*√*
Tacrolimus levels^b^							*√*	*√*
Tacrolimus dose adjustment				*√* ^ c^	*√* ^ c^	*√* ^ c^		
Adverse events		*√*	*√*	*√*	*√*	*√*	*√*	
Efficacy assessment								
EDSS		*√*					*√*	
MSFC		*√*					*√*	
FAMS		*√*					*√*	
VAS		*√*					*√*	
MRI		*√*					*√* ^ e^	

^a^If not done in the last 30 days or if washout period from prohibited medications is required.

^b^At week 8, tacrolimus levels will be obtained predose and 1.5 hours after the dose.

In the event that a change to a chronic medication is needed, tacrolimus blood levels will be obtained within 7–14 days if the concomitant medication is known to interfere with the metabolism of tacrolimus.

^c^If necessary, and at any time during the study period.

^d^Should the patient require a longer period to titrate the administration of interferon beta-1b, visit 3 can be delayed until after the patient has been on a full dose of interferon beta-1b for at least 1 month.

^e^In the event of early discontinuation, MRI will be performed at final visit if patient completed at least 3 months of treatment on the combined therapy.

**Table 2:** Baseline patient characteristics.

	Low blood level	High blood level
	*n* = 12	*n* = 13
Age, median (range)	36.0 (29, 53)	42 (27, 50)
Male, *n* (%)	4 (33.3)	3 (23.1)
Relapsing-remitting MS, *n* (%)	7 (58.3)	11 (84.6)
Duration of disease prior to study, median (range)	4.0 (1.0, 5.0)	2.0 (1.0, 7.0)
EDSS in the last year, median (range)	3.5 (1.5, 6.5)	3.5 (1.0, 6.0)
EDSS in the last 2 years, median (range)	2.0 (0.0, 6.5)	2.0 (0.0, 5.0)
Relapses in the last year, median (range)	1.5 (0.0, 3.0)	2.0 (0.0, 5.0)
Relapses in the last 2 years, median (range)	2 (0.0, 5.0)	3.0 (1.0, 7.0)
Number of diseases modifying drugs, median (range)	1 (1, 2)	2 (1, 3)
Mitoxantrone received prior to enrollment, *n* (%)	0 (0.0)	1 (7.7)

**Table 3:** Adverse events.

Adverse event, *n* (%)	Low blood level	High blood level	Total	*P* value
	*N* = 12	*N* = 13	*N* = 25	
Injection site reaction	10	7	17	0.012
Headache	6	11	17	0.097
Influenza-like symptoms	5	9	14	0.238
Diarrhea	6	6	12	1.000
Upper respiratory tract infection	5	6	11	1.000
Tremor	4	7	11	0.428
Hypomagnesemia	1	10	11	0.001
Paraesthesia	4	6	10	0.668

## References

[1] Bar-Or A (2008). The immunology of multiple sclerosis. *Seminars in Neurology*.

[2] Boggild M (2006). Rationale and experience with combination therapies in multiple sclerosis. *Journal of Neurology*.

[3] Knobler RL, Greenstein JI, Johnson KP (1993). Systemic recombinant human interferon-*β* treatment of relapsing-remitting multiple sclerosis: pilot study analysis and six-year follow-up. *Journal of Interferon Research*.

[4] The IFNB Multiple Sclerosis Study Group (1993). Interferon beta-1b is effective in relapsing-remitting multiple sclerosis. I. Clinical results of a multicenter, randomized, double-blind, placebo-controlled trial. *Neurology*.

[5] Paty DW, Li DKB (1993). Interferon beta-1b is effective in relapsing-remitting multiple sclerosis. II. MRI analysis results of a multicenter, randomized, double-blind, placebo- controlled trial. *Neurology*.

[6] The IFNB Multiple Sclerosis Study Group and The University of British Columbia MS/MRI Analysis Group (1995). Interferon beta-1b in the treatment of multiple sclerosis: final outcome of the randomized controlled trial. *Neurology*.

[7] Spencer CM, Goa KL, Gillis JC (1997). Tacrolimus. An update of its pharmacology and clinical efficacy in the management of organ transplantation. *Drugs*.

[8] Schreiber SL, Crabtree GR (1992). The mechanism of action of cyclosporin A and FK506. *Immunology Today*.

[9] Tocci MJ, Matkovich DA, Collier KA (1989). The immunosuppressant FK506 selectively inhibits expression of early T cell activation genes. *Journal of Immunology*.

[10] Uchino H, Minamikawa-Tachino R, Kristián T (2002). Differential neuroprotection by cyclosporin A and FK506 following ischemia corresponds with differing abilities to inhibit calcineurin and the mitochondrial permeability transition. *Neurobiology of Disease*.

[11] Woo J, Ross CSK, Milton JI, Thomson AW (1990). Immunosuppressive activity of FK-506 in rats; flow cytometric analysis of lymphocyte populations in blood, spleen and thymus during treatment and following drug withdrawal. *Clinical and Experimental Immunology*.

[12] Woo J, Sewell HF, Thomson AW (1990). The influence of FK-506 and low-concentration ciclosporin on human lymphocyte activation antigen expression and blastogenesis: a flow cytometric analysis. *Scandinavian Journal of Immunology*.

[13] Sharkey J, Jones PA, McCarter JF, Kelly JS (2000). Calcineurin inhibitors as neuroprotectants: focus of tacrolimus and cyclosporin. *CNS Drugs*.

[14] Gold BG, Voda J, Yu X, McKeon G, Bourdette DN (2004). FK506 and a nonimmunosuppressant derivative reduce axonal and myelin damage in experimental autoimmune encephalomyelitis: neuroimmunophilin ligand-mediated neuroprotection in a model of multiple sclerosis. *Journal of Neuroscience Research*.

[15] Wang MS, Gold BG (1999). FK506 increases the regeneration of spinal cord axons in a predegenerated peripheral nerve autograft. *Journal of Spinal Cord Medicine*.

[16] Wang MS, Zeleny-Pooley M, Gold BG (1997). Comparative dose-dependence study of FK506 and cyclosporin A on the rate of axonal regeneration in the rat sciatic nerve. *Journal of Pharmacology and Experimental Therapeutics*.

[17] Yoshikawa H, Iwasa K, Satoh K, Takamori M (1997). FK506 prevents induction of rat experimental autoimmune myasthenia gravis. *Journal of Autoimmunity*.

[18] McMichael J, Lieberman R, McCauley J, Irish W, Marino I, Doyle H (1996). Computer-guided randomized concentration-controlled trials of tacrolimus in autoimmunity: multiple sclerosis and primary biliary cirrhosis. *Therapeutic Drug Monitoring*.

[19] PROGRAF prescribing information.

[20] McDonald WI, Compston A, Edan G (2001). Recommended diagnostic criteria for multiple sclerosis: guidelines from the International Panel on the Diagnosis of Multiple Sclerosis. *Annals of Neurology*.

[21] BETASERON Prescribing information.

